# Editorial: Repurposing cancer immunotherapies for use in autoimmunity and transplantation

**DOI:** 10.3389/fimmu.2025.1690655

**Published:** 2025-09-01

**Authors:** Dominic A. Boardman, Lesley A. Smyth, Leonardo M.R. Ferreira

**Affiliations:** ^1^ Department of Surgery, The University of British Columbia, Vancouver, BC, Canada; ^2^ BC Children’s Hospital Research Institute, Vancouver, BC, Canada; ^3^ School of Medicine and Biosciences, University of West London, Ealing, United Kingdom; ^4^ Department of Pharmacology and Immunology, Medical University of South Carolina, Charleston, SC, United States; ^5^ Hollings Cancer Center, Medical University of South Carolina, Charleston, SC, United States

**Keywords:** cancer immunotherapy, immune tolerance, autoimmune disease, organ transplant rejection, graft-vs-host disease, chronic inflammation, regulatory T cells (Tregs), immune receptor

Cancer immunotherapy has reshaped modern medicine, providing new hope to patients by mobilizing the immune system to eliminate malignancies with potency and precision. While these therapies were originally designed to enhance immune responses against tumors, recent years have seen a transformative shift in the application of their underlying technology, with many of these pro-inflammatory and cytotoxic tools now being repurposed in a new array of diseases to achieve the opposite effect: immune tolerance. This Research Topic, *Repurposing Cancer Immunotherapies for Use in Autoimmunity and Transplantation*, brings together a Research Topic of pioneering studies and reviews offering proof-of-concept studies, new mechanistic insights, and translational outlooks on how lessons learned from immuno-oncology are being repurposed to suppress, rather than stimulate, the immune system to establish tolerance in autoimmune disease, organ transplant rejection, graft-vs-host disease, and chronic inflammation.

## Reprogramming Tregs: targeting alloreactive B cells and lymphoid niches

Regulatory T cells (Tregs), a subset of CD4^+^ T cells dedicated to inhibiting immune responses and maintaining immune homeostasis, hold great promise as living therapeutics to establish immune tolerance in organ transplantation, graft-vs-host disease, and autoimmune disease (1, 2). Two studies in this Research Topic underscore the versatility of Tregs when armed with cancer therapy-inspired engineering strategies. One, by Ferreira and colleagues, introduces chimeric anti-HLA antibody receptor (CHAR) Tregs designed to suppress alloantigen-specific B cells in HLA sensitized transplant recipients (Valentin-Quiroga et al.). This elegant approach transforms Tregs into highly selective suppressors of anti-HLA-A2 B cells by equipping them with a synthetic receptor to recognize and inhibit these pathogenic antibody-producing cells. Importantly, CHAR Tregs remained non-cytotoxic and suppressed antibody production by HLA-A2 sensitized patient cells exposed to HLA-A2. This specificity could dramatically improve desensitization protocols in transplant medicine and extend the lifetime of organ transplants, moving beyond broad immunosuppression to precision targeting of harmful immune subsets. In the second study, Fousteri, Bonini, Biswas, and colleagues present CXCR5-engineered Tregs as a strategy to improve their localization and function in secondary and tertiary lymphoid tissues, aspects often overlooked in engineered cell therapies (Doglio et al.). In humanized mice, human Tregs co-expressing CXCR5 and an HLA-A2-specific chimeric antigen receptor (CAR) (3–5) trafficked to and persisted in transplanted HLA-A2 positive human pancreatic islets without impairing islet function, in line with previous studies (6). Similarly, mouse Tregs co-expressing CXCR5 and a T cell receptor fusion construct (TRuC) against FVIII (7) displayed improved homing to and persistence in the spleen and lymph nodes, and suppressed anti-drug antibody (ADA) responses to recombinant FVIII protein to a greater extent than control FVIII TRuC Tregs in immunocompetent mice. These studies not only highlight the flexibility of Treg cell therapy but also emphasize the importance of molecular targeting, a key principle learned from CAR T cell therapy in oncology.

## Forging the path to precision modeling of autoimmune disease

Type 1 diabetes (T1D) is driven by the targeted immune destruction of insulin-producing β-cells, with CD8^+^ T cells playing a central pathogenic role. However, efforts to study these autoreactive T cells have been stymied by their rarity in the peripheral blood (8). Brusko and colleagues address this challenge through innovative use of CRISPR/Cas9-mediated genome editing and lentiviral vector technology to reprogram the antigen specificity of primary human CD8^+^ T cells (Peters et al.). By targeting the endogenous TCRα gene (TRAC) locus, these researchers generated HLA-A2-restricted islet peptide-specific CD8^+^ T cells that were cytotoxic towards HLA-A2 positive β-cells and secreted inflammatory cytokines. This work represents important progress not only on our ability to model autoimmunity more precisely, but also on preventing or even reversing it.

## Repurposing cancer drugs for autoimmune diseases

Cancer therapeutics often exploit vulnerabilities in rapidly dividing cells – a characteristic shared by autoreactive immune cells. AZD6738, an ATR kinase inhibitor originally developed for oncologic indications (9), is now being investigated for T1D prevention. As reported in this Research Topic by Sugitani et al., short-term treatment with AZD6738 prevents T1D and delays its onset in non-obese diabetic (NOD) mice by selectively depleting highly proliferative, self-reactive T cells. This strategy, if finetuned, can preferentially target autoaggressive cells while preserving overall immune competence. Similarly, dual inhibition of PI3Kδ and PI3Kγ, kinases often hyperactivated in B cell malignancies, shows promise in autoantibody-driven diseases like lupus. Marshall and colleagues show that Duvelisib, a PI3Kδ/γ inhibitor (10), significantly reduced B cell activation and autoantibody production, with associated improvements in kidney pathology, in the TAPP1^R218L^xTAPP2^R211L^ PI3K pathway dysregulation-driven mouse model of lupus-like disease (Olayinka-Adefemi et al.). Together, these exciting results suggest that drugs designed to inhibit tumor growth can be redirected to selectively eliminate pathogenic lymphocytes in autoimmunity.

## Towards a broader understanding of regulatory cell therapy

The field of regulatory cell-based therapies is rapidly expanding. Three review articles in this Research Topic provide critical frameworks for understanding the diversity of regulatory immune cells, progress using them in the clinic, and the context-dependent behavior of immunomodulatory signals. One review by Ferreira, Aref, and colleagues examines the therapeutic potential of Tregs, regulatory B cells (Bregs), tolerogenic dendritic cells, and myeloid-derived suppressor cells (Ghobadinezhad et al.). Each have demonstrated suppressive activity in preclinical autoimmune disease models and represents a modular tool for tuning immune responses, with efforts using Tregs being the furthest along in the clinic. As our understanding of Treg biology has deepened, so too has the ambition to translate this knowledge into the clinic. A review by Bluestone et al. provides a comprehensive and authoritative overview of Treg biology and the development of Tregs as living therapeutics by academic hospital centers and biotechnology companies, outlining both the promise and the remaining hurdles of developing Treg-based interventions into a third pillar of medicine, alongside conventional drugs and biologics. Another review, by Skartsis et al., examines the paradoxical effects of tumor necrosis factor alpha (TNFα) signaling. Often viewed as a proinflammatory cytokine, named after its ability to kill tumor cells (11), TNFα also plays roles in immune homeostasis and regulatory T cell function (12). The dual roles of membrane-bound versus soluble TNFα, along with differential tumor necrosis factor receptor TNFR1 and TNFR2 signaling pathways, suggest that nuanced targeting will be key to exploiting TNFα pathways therapeutically.

## The path forward: from oncology to immune tolerance

The contributions in this Research Topic collectively paint a compelling picture: cancer immunotherapies can be elegantly and efficaciously inverted and redirected to promote immune tolerance. The transition from tumor eradication to immune modulation is not just a lateral move. It is a profound shift that requires careful consideration of context, cell types involved, and antigen specificity. Cancer therapies have driven tremendous progress in precision T cell engineering, immune signaling network manipulation, and cell survival pathway targeting. These same strategies, reimagined through the lens of immune tolerance, are now fueling innovation in transplantation and autoimmunity. Nevertheless, challenges remain. Stability of engineered cells, tissue targeting efficiency and persistence, potential for unwanted off-target effects, and epitope spreading (which can be seen as autoimmunity’s counterpart to cancer’s immune editing) are all hurdles shared across oncology and autoimmunity. But with continued collaboration across fields and an ever-growing toolkit of synthetic biology, gene editing, and cell therapy strategies, the future looks promising. We are at a critical inflection point in immune therapy. The same ingenuity that gave rise to checkpoint inhibitors, CAR T cell therapy, and precision medicine cancer drugs is now being directed towards building a new class of tolerance-inducing therapies for autoimmunity, transplant rejection, graft-vs-host disease, allergies, and inflammatory disorders. We hope this Research Topic inspires continued basic research, clinical trials, and interdisciplinary collaboration to bring these therapies to the patients who desperately need them.
